# Pheochromocytoma and Diffuse Large B‐Cell Lymphoma in the Ipsilateral Adrenal Gland: A Case Report

**DOI:** 10.1002/iju5.70118

**Published:** 2025-11-10

**Authors:** Ken Maekawa, Toru Sakatani, Yuki Kita, Kimihiko Masui, Takayuki Goto, Yuki Teramoto, Taisuke Hosokai, Ichiro Yamauchi, Sho Koyasu, Takashi Kobayashi

**Affiliations:** ^1^ Department of Urology Kyoto University Hospital Kyoto Japan; ^2^ Department of Diagnostic Pathology Kyoto University Hospital Kyoto Japan; ^3^ Department of Clinical Oncology Kyoto University Hospital Kyoto Japan; ^4^ Department of Diabetes, Endocrinology and Nutrition Kyoto University Hospital Kyoto Japan; ^5^ Department of Diagnostic Radiology Kyoto University Hospital Kyoto Japan

**Keywords:** adrenal collision tumor, CVD chemotherapy, malignant lymphoma, pheochromocytoma

## Abstract

**Introduction:**

Pheochromocytoma is a catecholamine‐producing tumor arising from the adrenal medulla. When it coexists with a tumor of different origin within the same adrenal gland, it is classified as a collision tumor involving a pheochromocytoma.

**Case Presentation:**

The left adrenal tumor was identified in an 84‐year‐old Japanese woman and initially considered pheochromocytoma; however, rapid growth, lymphadenopathy, reduced ^123^I‐MIBG uptake, and intense FDG (Fluorodeoxyglucose) accumulation raised suspicion of another tumor component. The patient received cyclophosphamide, vincristine and dacarbazine (CVD) chemotherapy followed by surgical resection. Histopathology revealed extensive necrosis in the diffuse large B‐cell lymphoma (DLBCL) component, suggesting a response to chemotherapy.

**Conclusion:**

To the best of our knowledge, this is the fourth reported case of an adrenal collision tumor with pheochromocytoma and DLBCL, and the first treated with CVD followed by surgery. Collision tumors should be considered a differential diagnosis when adrenal masses present with atypical clinical or imaging features.

**Abbreviations**
^68^Ga‐DOTATOC‐PET: Gallium‐68 DOTA‐D‐Phe^1^‐Tyr^3^‐octreotide positron emission tomography
^123^I‐MIBG: iodine‐123 metaiodobenzylguanidine
CT: computed tomography
CVD: cyclophosphamide, vincristine, dacarbazine
DLBCL: diffuse large B‐cell lymphoma
FDG‐PET: fluorodeoxyglucose positron emission tomography
IL‐2R: interleukin‐2 receptor
MRI: magnetic resonance imaging
NSE: neuron‐specific enolase
PAL: primary adrenal lymphoma
PASS: pheochromocytoma of the adrenal gland scaled score
PPGL: pheochromocytoma and paraganglioma
PTHrP: parathyroid hormone‐related peptide
R‐CHOP: rituximab, cyclophosphamide, doxorubicin, vincristine, and prednisolone

**Summary**

- We report a rare case of pheochromocytoma coexisting with malignant lymphoma developed in the left adrenal gland, in which surgical resection of the primary tumor was performed following CVD chemotherapy.

## 1 Introduction

Pheochromocytoma is a catecholamine‐producing tumor arising from the adrenal medulla [1]. When pheochromocytoma coexists with another tumor of a different origin within the same adrenal gland, the condition is referred to as “collision tumor composed of pheochromocytoma,” which is rare [2]. Herein, we report a case of a collision tumor in the left adrenal gland, composed of a pheochromocytoma and diffuse large B‐cell lymphoma (DLBCL). To the best of our knowledge, this is the first reported case in which surgical resection was performed following cyclophosphamide, vincristine and dacarbazine (CVD) chemotherapy, making it a noteworthy contribution to the literature.

## 2 Case Report

**FIGURE 1:**
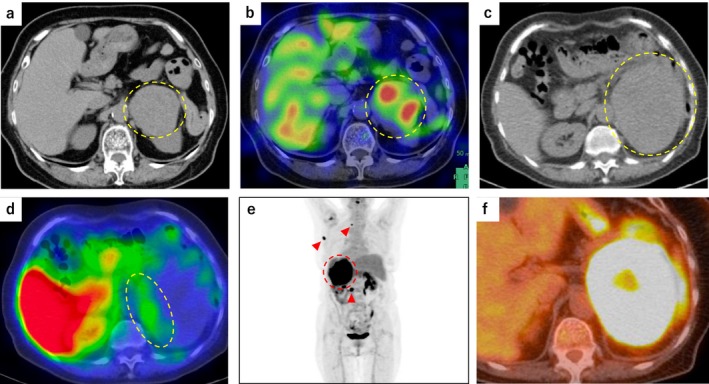
MIBG scintigraphy and FDG‐PET/CT findings. (a, b) At diagnosis, a non‐contrast CT image (a) at the same axial level as the MIBG scintigraphy image (b) shows a left adrenal mass with MIBG uptake (yellow dotted circle). (c, d) Four months later, a non‐contrast CT image (c) corresponding to the MIBG scintigraphy slice (d) shows the size of the left adrenal mass, while MIBG uptake is decreased but partially retained (yellow dotted circle). (e) FDG‐PET/CT maximum intensity projection (MIP) image shows intense FDG uptake in the left adrenal mass (red dotted circle) and newly developed lymphadenopathy in the para‐aortic, left axillary, and left supraclavicular regions (red arrowheads). (f) Axial FDG‐PET/CT image demonstrates intense FDG uptake in the left adrenal mass.

An 84‐year‐old Japanese woman was referred to a previous hospital after a left adrenal mass was incidentally detected on computed tomography (CT) during routine health screening (Figure 1a). She was asymptomatic, and had a normal heart rate and well‐controlled blood pressure by taking amlodipine. Physical examination revealed a painless mass in the left abdomen. Biochemical tests showed elevated plasma norepinephrine (4001 pg/mL). Iodine‐123 metaiodobenzylguanidine (^123^I‐MIBG) scintigraphy demonstrated intense uptake in the left adrenal mass, consistent with pheochromocytoma (Figure 1a,b).

Follow‐up whole‐body CT and abdominal magnetic resonance imaging (MRI) performed 3 months later showed further enlargement of the adrenal mass and newly developed lymphadenopathy involving the para‐aortic, left axillary, and left supraclavicular regions. Based on these findings, the patient was referred to our institution for further evaluation and management. On initial evaluation, laboratory data revealed elevated serum lactate dehydrogenase (LDH, 1017 U/L), neuron‐specific enolase (NSE, 56.6 ng/mL), parathyroid hormone‐related peptide (PTHrP, 2.9 pmol/L) and soluble interleukin‐2 receptor (sIL‐2R, 1750 U/mL). Plasma norepinephrine remained elevated (1070 pg/mL), as did 24‐h urinary norepinephrine (419 μg/day) and normetanephrine (0.93 mg/day).

Repeat ^123^I‐MIBG scintigraphy performed 4 months after the initial diagnosis showed decreased uptake in the left adrenal mass (Figure 1c,d), with no uptake in the enlarged lymph nodes. In contrast, ^18^F‐fluorodeoxyglucose (FDG) positron emission tomography/computed tomography (PET/CT) revealed intense FDG uptake in both the adrenal mass and all enlarged lymph nodes (Figure 1e,f), raising the suspicion of dedifferentiation and high‐grade malignancy of the pheochromocytoma with nodal metastases.

CVD chemotherapy was initiated. After two cycles, imaging demonstrated tumor regression in both the adrenal lesion and the lymphadenopathy. Gallium‐68 DOTA‐D‐Phe^1^‐Tyr^3^‐octreotide positron emission tomography (^68^Ga‐DOTATOC‐PET) was subsequently performed in anticipation of internal radiotherapy, but no uptake was observed in the lesions. Following three CVD cycles, the adrenal mass shrank from 12 to 6.5 cm, para‐aortic lymph nodes from 1.6 to 0.5 cm, and other lymphadenopathies resolved completely. The patient underwent open resection of the retroperitoneal tumor and para‐aortic lymphadenectomy via a chevron incision in the supine position. Operative time was 4 h 8 min, with an estimated blood loss of 200 mL, and intraoperative blood pressure remained stable throughout the procedure.

**FIGURE 2:**
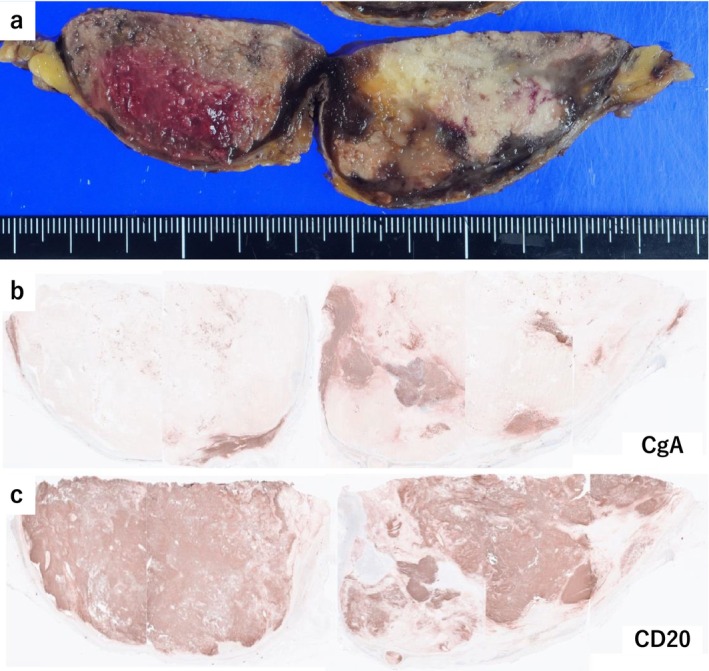
Gross and immunohistochemical findings of the left adrenal tumor. (a) Gross appearance of the cut surface. (b) Chromogranin A (CgA) immunostaining indicating the distribution of pheochromocytoma. (c) CD20 immunostaining highlighting the distribution of diffuse large B‐cell lymphoma.

**FIGURE 3:**
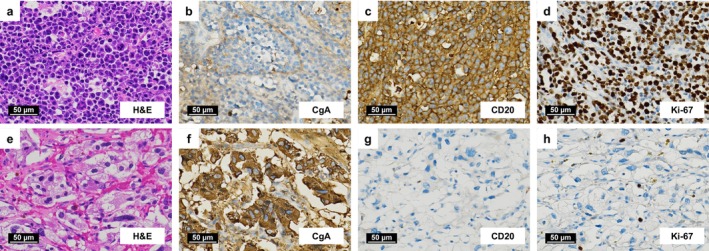
Histopathological and immunohistochemical findings. (a–d) Diffuse large B‐cell lymphoma. (e–h) Pheochromocytoma. (a, e) Hematoxylin and eosin (H&E) staining. (b, f) Chromogranin A (CgA). (c, g) CD20. (d, h) Ki‐67.

The resected tumor showed macroscopic heterogeneity, with a peripheral brown area and central necrosis (Figure 2a). Histologically, the brown rim corresponded to pheochromocytoma, which was positive for chromogranin A (Figure 2b) and had a PASS of 2, suggesting low malignant potential. In contrast, the bulk of the tumor consisted of CD20‐positive, chromogranin A‐negative cells (Figure 2b,c) with a Ki‐67 labeling index of approximately 90%, consistent with diffuse large B‐cell lymphoma (DLBCL; Figure 3). Most of the CD20‐positive area showed necrosis. No involvement was found in the para‐aortic lymph node.

Based on these findings, the tumor was diagnosed as an adrenal collision tumor composed of pheochromocytoma and DLBCL. CVD therapy was mainly effective for the DLBCL component. The patient was discharged on postoperative day 15, with normalized catecholamine levels. Follow‐up FDG‐PET/CT performed 45 days after surgery showed no residual disease. A rituximab‐containing chemotherapy regimen was initiated thereafter.

## 3 Discussion

Collision tumors are defined as distinct neoplasms with independent origins and without tissue admixture, whereas composite tumors are characterized by an intermingling of neoplastic cells that share a common origin and driver mutation [3]. Based on this classification, the present case represents a collision tumor composed of pheochromocytoma and DLBCL in the left adrenal gland. The pathogenesis of such collision tumors remains unclear, but it is hypothesized that local immunologic or microenvironmental changes within the adrenal gland may facilitate the development of a second neoplasm, particularly in the context of chronic inflammation or immunosuppression [3]. To date, only three similar cases have been reported, highlighting the rarity of this condition [2, 4, 5]. To our knowledge, this is the first reported case of such a tumor treated preoperatively with a CVD regimen (Table 1). Cyclophosphamide and vincristine, components of this regimen, are also included in the standard rituximab, cyclophosphamide, doxorubicin, vincristine, and prednisolone (R‐CHOP) regimen for malignant lymphoma [6]. In this case, extensive necrosis in the DLBCL component of the resected tumor suggests that the CVD regimen was effective primarily against the lymphoma. While this case involved malignant lymphoma affecting the left adrenal gland, we do not classify it as primary adrenal lymphoma (PAL). However, we note that when PAL is suspected, early diagnosis and rituximab‐based chemotherapy are critical due to its generally poor prognosis [7, 8].

**TABLE 1:** Patient characteristics of mixed pheochromocytoma with malignant lymphoma.

No.	Author	Year	Age	Gender	Image	Size (cm)	Location	Biopsy	Chemotherapy	Surgery
1	A. Babinska	2015	57	Male	CT	13	Right adrenal gland	None	CHOP	Open adrenalectomy
2	A. Khorsand	2018	63	Male	Ultrasound	5	Right adrenal gland	None	Not described	Open adrenalectomy
3	O. H. Oraibi	2019	79	Male	CT	17	Left adrenal gland	None	Not described	Open adrenalectomy
4	The present case	2025	84	Female	CT, MRI, MIBG, FDG‐PET	12	Left adrenal gland	None	CVD	Open adrenalectomy

Abbreviations: CHOP, cyclophosphamide doxorubicin vincristine and prednisolone; CT, computed tomography; CVD, cyclophosphamide, vincristine and dacarbazine; FDG‐PET, fluorodeoxyglucose positron emission tomography; MRI, magnetic resonance imaging, MIBG, metaiodobenzylguanidine.

The left adrenal mass was initially diagnosed as a pheochromocytoma. However, 3 months later, the patient developed rapid tumor growth and lymphadenopathy. The enlargement of the mass decreased ^123^I‐MIBG uptake, and intense FDG accumulation raised the suspicion of dedifferentiation of pheochromocytoma, complicating the diagnosis. A retrospective review of the ^123^I‐MIBG scan performed 4 months after the initial diagnosis revealed both high‐ and low‐uptake areas within the adrenal mass, potentially indicating the coexistence of multiple tumor components (Figure 1d).

Although histological diagnosis is essential when suspecting a collision tumor composed of pheochromocytoma and malignant lymphoma, percutaneous needle biopsy of pheochromocytoma or paraganglioma (PPGL) carries risks of catecholamine crisis and hemorrhage [9]. However, according to Zhang et al., among 86 PPGLs, only a few developed catecholamine‐related complications after biopsy, with no reported mortality [9]. Thus, biopsy may be feasible in select cases with proper preparation and monitoring. In the present case, axillary lymph node biopsy may have been a viable diagnostic approach.

This case highlights the importance of considering the possibility of concurrent tumors, including malignant lymphoma, in the same adrenal mass as pheochromocytoma.

## 4 Conclusion

We report a rare case of a collision tumor comprising pheochromocytoma and malignant lymphoma within the same adrenal gland. Clinicians should consider the possibility of concurrent tumors in adrenal masses, especially with atypical clinical or imaging findings.

## Ethics Statement

This study was approved by the Institutional Review Board of Kyoto University Graduate School of Medicine (G0052‐17) and adhered to the Declaration of Helsinki.

## Consent

The patient provided written informed consent.

## Conflicts of Interest

The authors declare no conflicts of interest.

## Supporting information


**Figure S1:** S‐100 immunohistochemistry highlighting sustentacular cells. Immunostaining for S‐100 protein demonstrates the presence of sustentacular cells surrounding the nests of tumor cells, supporting the diagnosis of pheochromocytoma.

## Data Availability

Data sharing not applicable to this article as no datasets were generated or analyzed during the current study.
