# Supplementary figures and images for: Pheochromocytoma and Diffuse Large B‐Cell Lymphoma in the Ipsilateral Adrenal Gland: A Case Report
Source: IJU Case Rep. 2025 Nov 10;9(1):e70118. doi: 10.1002/iju5.70118 (PMC12747783; doi:10.1002/iju5.70118)

## Slide 1
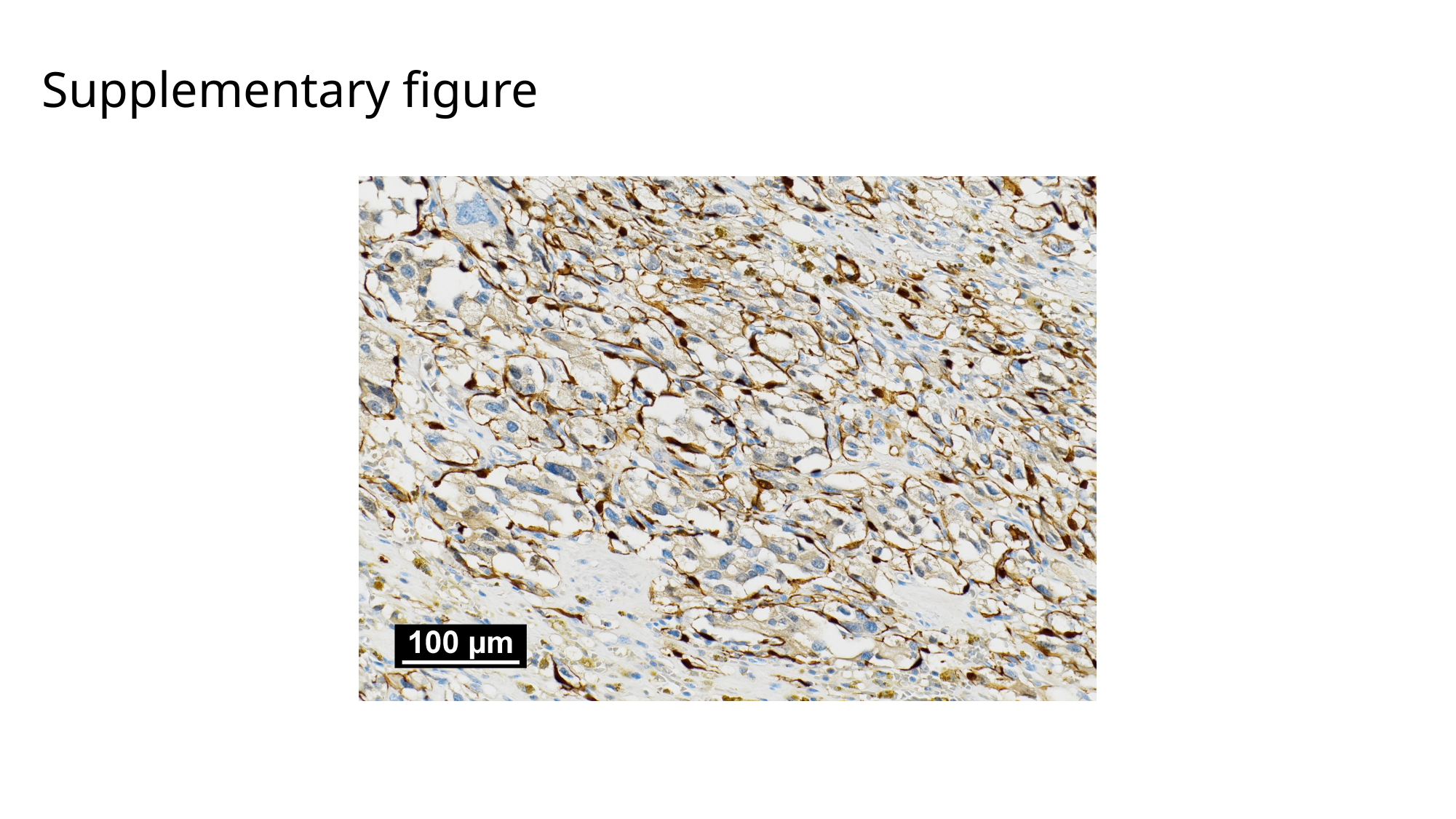

Supplementary figure

Supplement: Supplementary file 1 — Figure S1: S‐100 immunohistochemistry highlighting sustentacular cells. Immunostaining for S‐100 protein demonstrates the presence of sustentacular cells surrounding the nests of tumor cells, supporting the diagnosis of pheochromocytoma. [file IJU5-9-e70118-s001.pptx]
